# Empowering American Indian and Alaska Native youth to lead measurement development of an indigenous adolescent wellbeing measure: A protocol paper

**DOI:** 10.3389/fpubh.2022.994434

**Published:** 2022-11-18

**Authors:** Sierra Quintana, Jerreed D. Ivanich, Kimberly Pikok, Shanoa Nez, Zenetta Zepeda

**Affiliations:** ^1^Centers for American Indian and Alaska Native Health, University of Colorado Anschutz Medical Campus, Aurora, CO, United States; ^2^International Arctic Research Center, University of Fairbanks, Fairbanks, AL, United States; ^3^Mel and Enid Zuckerman College of Public Health, University of Arizona, Tuscon, AZ, United States; ^4^Biology Department, University of Colorado Denver, Denver, CO, United States

**Keywords:** American Indian and Alaska Native, youth led research, wellbeing, health, indigenous youth, indigenous health

## Abstract

**Background:**

American Indian and Alaska Native youth research has rarely included young people from within these populations as co-designers. In addition to the lack of youth involvement, most findings focus on presenting statistics around disparity vs. focusing on this population's unique strengths and resiliency. The research design of this protocol aims to fill this gap in the current literature.

**Methods:**

To address this discrepancy, a multipronged approach to youth and young adult participatory research was implemented. These prongs included a virtual gathering where the Nominal Group Technique was conducted and an assembly of a Youth Research Design Team. Lastly, the research team will implement a protocol developed by the Research Design Team. The Research Design Team plans to conduct qualitative interviews and distribute a web-based quantitative survey with a raffle as respondent compensation. This protocol is a preliminary phase to developing a wellbeing measure for AIAN youth.

**Discussion:**

Having an operationalized definition of wellness from AIAN youth will fill a gap in the current body of research with optimism that this will lead to additional studies exploring the AIAN youth voice.

## Introduction

Since the Indian Health Services was established in 1955, American Indian and Alaska Native Health (AIAN) research has focused primarily on addressing health disparities and less on cultural strengths and community perspectives on wellness (1). More research needs to be done to define what thriving looks like for this population in order to more effectively address health disparities. Engaging AIAN youth with deep cultural insights on the foundational factors of what it means to thrive can act as the framework to make sustainable and effective impacts. To achieve intervention goals relevant to the population being served, a clear understanding of what outcomes are meaningful to the individuals within that population is essential.

AIAN youth wellness research lacks strengths-based and AIAN-engaged approaches to health and wellness. Most AIAN youth wellness research focuses on suicide (2, 3) or diabetes (2, 4, 5) prevention. Scarcer than strengths-based AIAN wellness research is youth-led measures focused on defining and assessing wellbeing for AIAN youth. The AIAN youth-led wellness research focuses on a disparity and has youth help either work to identify causes (6, 7) or create an intervention to address an issue identified before youth were integrated into the project (8, 9). This study intends to ground our research in AIAN youth narratives that emphasize community strengths and connection to AIAN culture (1, 10). This initial phase seeks to inform the creation of a measure of AIAN wellbeing that is not in response to disparity but instead a measure of strengths.

Historically, AIAN youth wellness has been done by researchers from outside the community. This research focused primarily on implementing and evaluating western ideas of wellness, building on existing scientific wellness knowledge, and increasing publications on an often-neglected population (11). AIAN communities' high rates of poverty, mental health struggles, and poor health outcomes triggered by remnants of settler colonialism (1, 2, 12), cultural eradication (13, 14), and forced assimilation (15) are the justification for most AIAN research but rarely are the interventions focused on cultural honor, celebration or revitalization. The aims targeted in these studies were less about community prosperity and instead sought outcome improvements aligned with Western expectations. Results likely reflect the AIAN community's incompatibility to perform well when measured in a grid incompatible with their cultural practices and lifestyles (1). Other scholars, such as Fok et al. (16), have gone to great lengths to identify, create, and validate measures based on deep community feedback (16).

Youth-led, youth-directed, and youth participatory action research (YPAR) have shown promising results with many marginalized communities (4, 17–23). Ozer defines YPAR as “an approach to scientific inquiry and social change grounded in principles of equity that engages young people in identifying problems relevant to their own lives, conducting research to understand the problems, and advocating for changes based on research evidence (19).” Applying these research approaches to AIAN people has yet to be done to create a measure that assesses positive attributes of this population.

Our protocol aims to fill this gap in research. This protocol is currently in progress and will inform phase two, which includes developing and validating the full AIAN youth wellbeing measure. When this manuscript was submitted for publication, two of three prongs were completed. Our protocol takes a multipronged approach to engage AIAN youth to inform and direct our research. First, we convened a group of AIAN youth in the Nominal Group Technique (NGT) experience. Second, we assembled a YPAR AIAN youth Research Design Team to develop another component of data collection that will make up the third and final prong. The preliminary data collection and analysis of phase one intends to lay the foundation for an eventual wellbeing measure that reflects how AIAN youth define wellness. The youth's involvement aims to incorporate AIAN cultural elements from the perspective of young people's life experiences as an AIAN person in today's society.

In our first prong, we engaged AIAN youth using the NGT. The NGT is a consensus technique developed in the 1970s by researchers Delbecq and Van De Ven (24) to create more collaborative adult education programing (24–28). This method emphasizes the opportunity for each participant's ideas and opinions to be heard without criticism. It comprises four components: silent generation of ideas, round-robin sharing, clarification, and a ranking of contributions (25). This method has been used with many other underserved communities (28–32) but has yet to have publications exploring it is use with AIAN populations.

We recruited and convened a three-person AIAN Youth Research Design Team in our second prong. This Research Design Team will remain as contributors to the project through all phases. They were and will continue to be central contributors to the development, data collection, and findings dissemination for this project from the team's launch to the project's conclusion. They will lead the research team in designing the third prong of AIAN engagement which will include another component of data collection and methods to optimize findings dissemination to AIAN youth. These components aim to centralize AIAN youth voices and allow for their input and direction at each development step.

##  Methods

### Recruitment and setting

For all aspects of this protocol, participation criteria were: 18–24 years of age, self-identifying member of an American Indian or Alaska Native tribe, and willing to sign a consent form for participation. Members of our recruitment pool groups live throughout the United States, representing a diverse number of tribes and ethnic backgrounds. Meetings were held *via* virtual videoconference *via* the Zoom platform. The research team collected no additional demographic information to assure confidentiality.

## Nominal group technique

### Recruitment

Recruitment components specific to the NGT included: Participants were recruited from a subset of the Native Youth Congress team and the American Indian and Alaska Native Affinity group involved with the Annie E Casey Foundation's Youth and Young Adult Wellbeing project. All participants opted into the NGT experience after each group leader identified and distributed the research team's meeting description. 12 diverse AIAN youth from multiple locations and culturally distinct AI/AN communities participated. Each participant received a $200 electronic gift card for attending the session.

### Nominal group technique protocol

**Figure 1:**
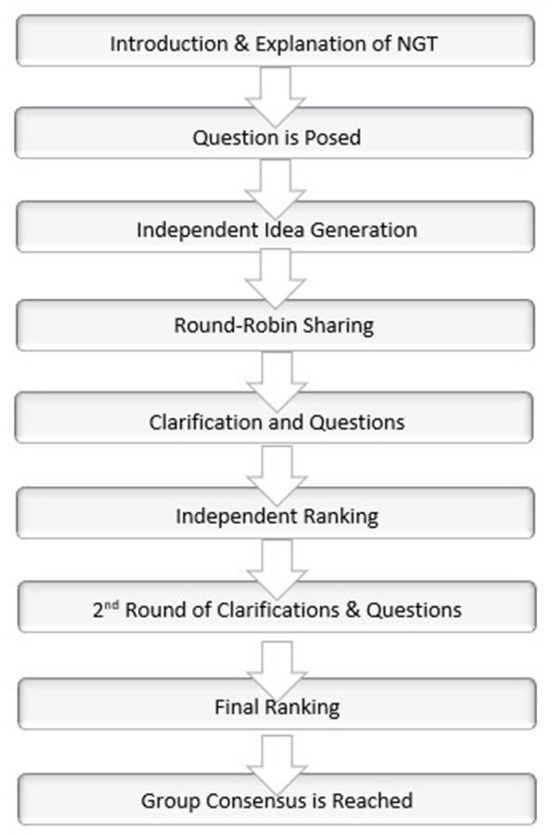
Nominal group technique (NGT) flowchart.

The Nominal Group Technique (Figure 1) was conducted as follows:

#### Introduction

Participants were welcomed, and the purpose of the meeting was explained as an opportunity to openly discuss a question where all ideas are valued and considered regardless of scope. Emphasis was put on idea generation and collaboration vs. finding one accurate solution.

#### Independent idea generation

The question posed was, “What is wellbeing for American Indian and Alaska Native Youth?” Participants were given seven minutes to silently brainstorm and list as many ideas as they could generate. Seven minutes of uninterrupted time allowed for focus, reflection, and mapping out ideas.

#### Round-robin sharing

A research team member convened the group, went around to each participant, and recorded one idea. The ideas were recorded on the Zoom whiteboard feature, visible to all meeting attendees, as presented by the participant, with no guidance from the research team. The research team continued to return to participants in the same order to contribute one idea per turn until all views were represented on the virtual whiteboard.

#### Clarification and questions

Once all ideas were listed, a research team member opened the floor for discussion. The group addressed each concept one at a time, as listed on the virtual whiteboard. Any participant or facilitator could ask questions for clarity, make suggestions to further develop ideas, or conciseness. All participants could discuss disagreements about an idea and present arguments. The group discussed ideas objectively without further attachment to the original contributor. Finally, the group could combine ideas if two or more are identified as equivalent.

#### Initial ranking

After clarification and questions, the group narrowed with conciseness and elimination of duplicates. At this time, a research team member asked participants to choose roughly half the total number of ideas and rank them from most important to least important. At the beginning of the ranking process, the group had 15 ideas.

The research team asked each participant to give their top ideas a ranking value. There are eight ideas to rank, so each person has 8, 7, 6, 5, 4, 3, 2, and 1 ranking values to assign to each idea. Participants gave their top idea eight ranking values, their second most important idea, seven ranking value, and so on (1^st^ most important = 8 ranking value, 2^nd^ most important 7 ranking value, 3^rd^ = 6, 4^th^ = 5, 5^th^ = 4…). Participants then added their ranking values to the virtual whiteboard.

These values were added, and the idea with the highest added ranking value became the number one answer for the question, the idea with the second-highest added ranking value becomes the second most agreed upon answer, and so on. The research team leads participants into the second discussion component once the top eight ideas are identified based on added ranking values.

#### Second round of discussion for clarifications and questions

Participants were asked to discuss the eight highest-ranked solutions (Table 1). The research team went down the list from highest to lowest ranking. New questions, debates, and idea development arose as ideas were narrowed down. Once this was done, a research team member announced the definitive answer chosen to answer the question. Any additional questions or concerns were welcomed for discussion at this point. At this time, a group consensus for answering the question was reached.

**Table 1:** Nominal group technique top eight answers.

**Rank**	**Ideas**
1	Cultural identity
2	Healthy relationships with family and friends
3	Physical safe spaces
4	Mental health
5	Nature is wellness (cultural aspects of the natural elements)
6	Traditional practices
7	Mentorship
8	Cultural community support

## Research design team assembly

A three-member AIAN youth Research Design Team was created with participants between 18 and 24 to lead design and aid in developing a measure that describes what wellbeing means to AIAN youth. The Research Design Team meets weekly with the research team to develop methods and data collection plans.

### Recruitment

All Research Design Team members were recruited *via* email from Principal Investigator (PI) community connections. Each potential participant was contacted *via* email and invited to a virtual interview where the PI and research team assessed fit for the project. Once suitability was confirmed, candidates were invited to join the project. The Research Design Team is comprised of one Alaska Native and two young American Indian adults. The three American Indian Research Design Team members come from various American Indian communities, including one that is urban-based.

### Compensation

Each Research Design Team member has been compensated with a stipend of $3,000 over 6 months distributed by the Aspen Institute.

## Research design team's protocol

In collaboration with the research team, the Research Design Team is in the progress of developing and implementing methods developed through weekly Research Design Team Meetings. This protocol includes a quantitative survey, five qualitative interviews, and community dissemination through social media.

### Platforms for data collection

Quantitative survey: The survey will be built in Qualtrics and distributed over Listservs and social media.

Qualitative interviews: All interviews will be conducted *via* virtual Zoom meetings.

Community dissemination: Clips of the qualitative interviews will be posted on social media sites such as TikTok, Instagram, and Facebook, with links sent out through the University of Colorado and Aspen Institute Listservs.

### Sample size

Quantitative survey: We aim to get 100 AIAN young adults to take the web-based survey.

Qualitative interviews: Five individuals will be selected for a qualitative interview.

Community dissemination: Due to the nature of social media, we have no specific sample size. We aim to reach as many viewers as possible.

### Recruitment

Quantitative survey: Recruitment will be conducted through social media posts (Instagram, TikTok, Facebook, etc.) and sent to the University of Colorado and Aspen Institute's Listservs.

Qualitative interviews: Five individuals will be randomly selected from survey respondents and invited to a qualitative interview.

Community dissemination: No recruitment is required.

### Compensation

The five individuals who agree to participate in the qualitative interviews will be given randomized respondent compensation, consisting of Native artwork valued between 200 and $250. The youth Research Design Team feels it is essential to support Native artists and creators and provide compensation that is outside of the typical gift card that would be more culturally relevant to the participants. While many western institutions, including the PIs academic institution, often do not have systems for this type of respondent compensation, our youth Research Design Team pushed for such compensation, and creative solutions were found to accommodate.

##  Discussion

The NGT is an effective method for capturing consensus among small groups (24–26). To develop a youth-directed operationalized definition of AIAN youth wellness, we plan to use the collaborative nature of the NGT and the community members' insights to understand and improve outcomes for this population.

**Figure 2:**
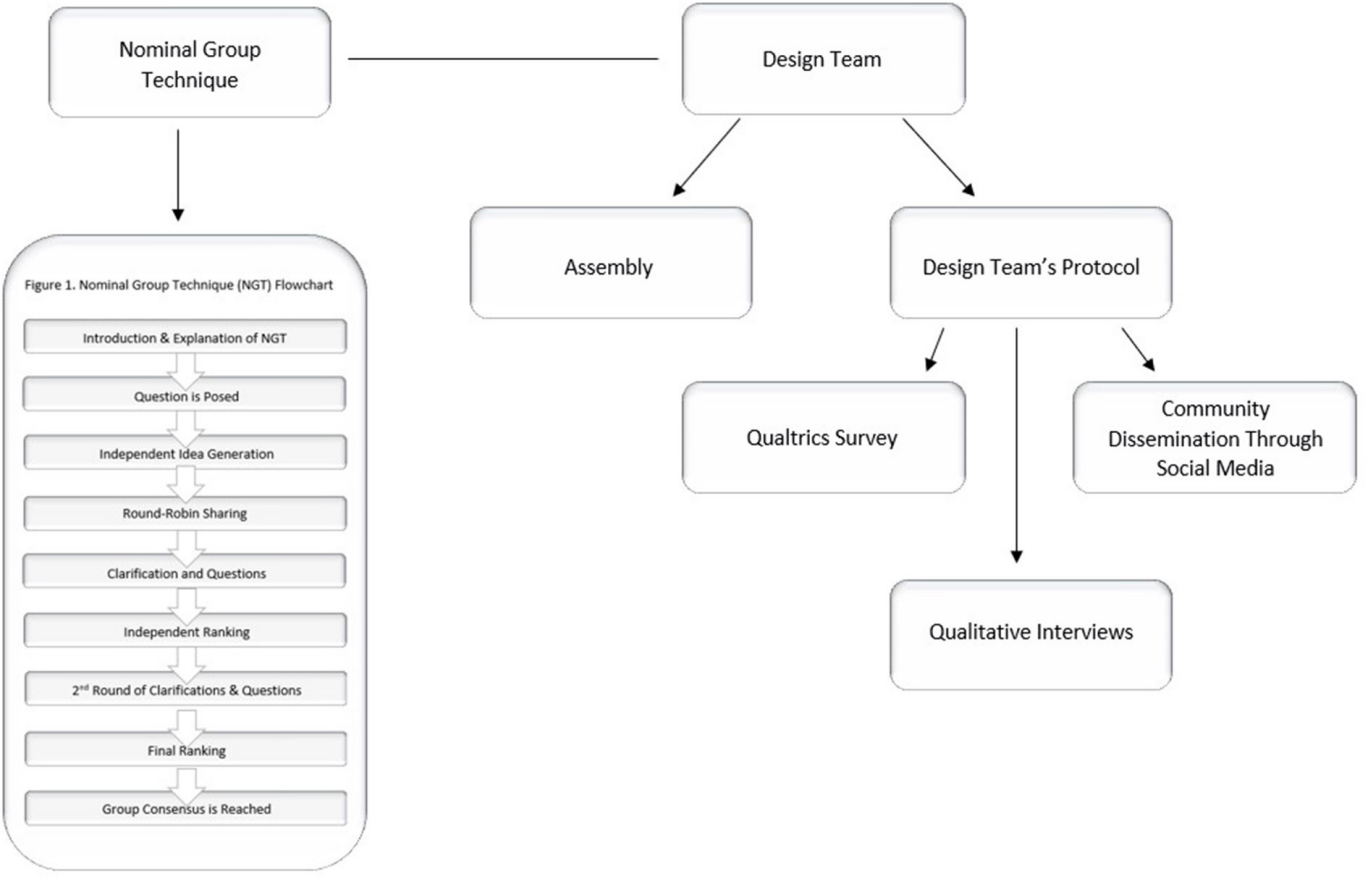
Project overview highlighting multipronged approach.

The addition of the Research Design Team allows for elements of research that come directly from AIAN youth. The data collected from these measures will provide comparative data to understand if the finding from the NGT echoes through larger portions of AIAN youth. We desire to empower young AIAN people, capture this population's current perspectives and voice, and hopefully inspire more AIAN scholars in the future. The Research Design Team's components lend a multipronged approach (Figure 2) to this study. They provide the research team with a more complex understanding of how AIAN youth define wellbeing with multiple different data types with relatively minimal effort and burden on AIAN communities.

These approaches move away from focusing on reservations or villages as problem areas, overwhelmed with health disparities (1), and toward research founded on AIAN community strengths and resilience. Having an operationalized definition of AIAN youth wellbeing will fill a gap in the current body of research with optimism that this will lead to additional studies exploring the AIAN youth voice and the cultural strengths within AIAN communities. Opening the door to more strengths-based, youth-led, culturally revitalizing research may effectively address well-documented disparities within this community.

This approach has several strengths. This includes recruiting from pools of candidates from various tribes, geographical locations, and cultural backgrounds. This allows for a diverse collection of thoughts lending to a multi-layered AIAN perspective. Although not likely to fully represent one tribe but will ideally resonate for many AIAN peoples. Using the NGT encourages participation from all attendees without giving increased time to more vocal personalities and ensuring contribution from more reserved participants. Another strength is the innovative nature of using NGT with AIAN youth, as this technique has not been implemented in this population. This project is overseen by Colorado Multiple Review Board IRB, to publish all findings to contribute to the current science.

Weaknesses of this approach include that with innovation comes inexperience. Although researchers are well versed in the NGT, without previous studies working to guide adaption for nuances found when implementing this technique with AIAN, we anticipate potential room for improvements to engage this population more adequately. And while this project was conducted with 12 diverse AIAN youth from multiple geographical locations and culturally distinct AI/AN communities, they are not representative of the diversity among all AIANs youth.

##  Conclusion

American Indian and Alaska Native youth's worldviews and value systems are understandably different from a western understanding of wellbeing. To harness the unique knowledge of these differences, it is imperative that research not only be attuned to American Indian and Alaska Native voices, but those voices need to be youth-led. The unique combination of being culturally specific and youth-led provides a backdrop for the field of wellbeing research to be pushed in a new direction. That new direction actively seeks out opportunities to amplify voices that are often silenced or ignored. This study protocol highlights that improving our understanding of wellbeing is not hampered by being culturally sensitive and youth-led. In fact, the opposite, we are learning more from this intersecting collaboration.

## Ethics statement

The studies involving human participants were reviewed and approved by the Colorado Multiple Institutional Review Board. The patients/participants provided their written informed consent to participate in this study.

## Author contributions

All authors listed have made a substantial, direct, and intellectual contribution to the work and approved it for publication.

## Funding

This project was funded by the Annie E. Casey Foundation (GA-2021-X7042).

## Conflict of interest

The authors declare that the research was conducted in the absence of any commercial or financial relationships that could be construed as a potential conflict of interest.

## Publisher's note

All claims expressed in this article are solely those of the authors and do not necessarily represent those of their affiliated organizations, or those of the publisher, the editors and the reviewers. Any product that may be evaluated in this article, or claim that may be made by its manufacturer, is not guaranteed or endorsed by the publisher.
